# Supplementary material for: Treatment Modalities for Angina with Non-Obstructive Coronary Arteries (ANOCA): A Systematic Review and Meta-Analysis
Source: J Clin Med. 2025 Jun 9;14(12):4069. doi: 10.3390/jcm14124069 (PMC12194334; doi:10.3390/jcm14124069)
Supplement: Supplementary file 1 [file jcm-14-04069-s001.zip › File S1.pdf]

Systematic Review

Treatment modalities for Angina with Non-Obstructive Coronary Arteries (ANOCA) patients

Overview	
Interface	**Pubmed**
Date of Search	21st November 2024
Number of results	2,717 (+ 133 compared to last search 21st July 2023)

(“Microvascular Angina” [Mesh]) OR (“Coronary vasospasm” [Mesh])

OR

“ANOCA [tiab]” OR “angina with non-obstructive coronary arteries [tiab]” OR “coronary syndrome X [tiab]” OR “coronary microvascular dysfunction [tiab]” OR “microvascular angina [tiab]” OR “vasospastic angina [tiab]” OR “angina X syndrome [tiab]” OR “angina pectoris with normal coronary arteriogram [tiab]” OR “angina pectoris with normal coronary angiogram [tiab]” OR “coronary vasospasm [tiab]” OR “coronary artery spasm [tiab]”

AND

(“therapeutics” [Mesh])

OR

“treatment [tiab]” OR “therapeutics [tiab]”

Search 1: ((((((((((((angina, microvascular[MeSH Terms]) OR (coronary vasospasm[MeSH Terms])) OR (ANOCA[Title/Abstract])) OR (angina with non-obstructive coronary arteries[Title/Abstract])) OR (coronary syndrome X[Title/Abstract])) OR (coronary artery dysfunction[Title/Abstract])) OR (microvascular angina[Title/Abstract])) OR (vasospastic angina[Title/Abstract])) OR (angina X syndrome[Title/Abstract])) OR (angina pectoris with normal coronary arteriogram[Title/Abstract])) OR (angina pectoris with normal coronary angiogram[Title/Abstract])) OR (coronary vasospasm[Title/Abstract])) OR (coronary artery spasm[Title/Abstract])) AND ((("therapeutics"[MeSH Terms]) OR (treatment[Title/Abstract])) OR (therapeutics[Title/Abstract])))

Overview	
Interface	**EMBASE**
Date of Search	21st November 2024
Number of results	8,600 (+ 935 compared to last search)

‘microvascular angina’/exp OR ‘coronary vasospasm’/exp

OR

‘microvascular angina*’:ab,ti,kw OR ‘coronary vasospasm*’:ab,ti,kw OR ‘ANOCA*’:ab,ti,kw OR ‘angina with non-obstructive coronary arteries*’:ab,ti,kw OR ‘coronary syndrome X*’:ab,ti,kw OR ‘coronary microvascular dysfunction*’:ab,ti,kw OR ‘vasospastic angina*’:ab,ti,kw OR ‘angina X syndrome*’:ab,ti,kw OR ‘angina pectoris with normal coronary angiogram*’:ab,ti,kw OR ‘coronary artery spasm*’:ab,ti,kw

AND

‘treatment’/exp OR ‘therapy’/exp

OR

‘treatment*’:ab,ti,kw OR ‘therapy*’:ab,ti,kw

Overview	
Interface	**Cochrane**
Date of Search	21st November 2024
Number of results	2,486

(“Microvascular Angina” [Mesh]) OR (“Coronary Vasospasm” [Mesh])

OR

(“microvascular angina”):ti,ab,kw OR (“vasospastic angina pectoris”):ti,ab,kw OR (“vasospastic angina”):ti,ab,kw OR (“normal coronary arteries”):ti,ab,kw

AND

(“Theapeutics” [Mesh]) OR (“Pharmaceutical Preparations” [Mesh])

OR

(treatment):ti,ab,kw OR (treatment effect):ti,ab,kw OR (therapeutic):ti,ab,kw OR (therapy):ti,ab,kw

Results: 8 Cochrane reviews, 2477 Trials, 1 editorial
