# Supplementary material for: Treatment Modalities for Angina with Non-Obstructive Coronary Arteries (ANOCA): A Systematic Review and Meta-Analysis
Source: J Clin Med. 2025 Jun 9;14(12):4069. doi: 10.3390/jcm14124069 (PMC12194334; doi:10.3390/jcm14124069)
Supplement: Supplementary file 1 [file jcm-14-04069-s001.zip › File S2.pdf]

File S2

Supplementary Table S1: Study characteristics remaining studies

First Author	Year	Study type	Follow-up duration	N	Mean age Intervention	Mean age control	Male (%)	Results
Alpha-blockers								
Rosen	1999	Prospective, randomised	10 days	28	54,4 ± 7,6	Not given	35,7	CFR (PET – MBF (ml/min*100g)): baseline 3,03 ± 1,19 vs. follow-up 2,99 ± 1,31 (p = ns)
Bøtker	1998	Prospective, randomised (cross-over)	20 weeks	16	56 ± 5	N/A	12,5	Exercise capacity (s): baseline 488 ± 185 vs. follow-up 489 ± 240 (p = ns)
Robertson	1983	Prospective, randomised (cross-over)	6 days	6	56,3 ± 13	N/A	50	Angina pectoris frequency (diary per day): baseline 4 ± 2 vs. follow-up 4 ± 2 (p = ns)
Sympathectomy								
Lin	2019	Prospective, randomised	24 months	79	51,8 ± 11,2	53,1 ± 9,3	56,9	All-cause mortality: sympathectomy group 2,7% vs. conventional group 14,3% (p = 0,0272)
L-arginine								
Palloshi	2004	Prospective, unrandomised	4 weeks	13	56 [48 – 66]	N/A	53,8	Angina pectoris frequency (diary per week): baseline 12 ± 3 vs. follow-up 4 ± 1 (p < 0,001)<br>Quality of life (visual analog scale): baseline 24 ± 19 vs. follow-up 72 ± 27 (p < 0,001)<br>CCS class: baseline 3,5 ± 1,1 vs. follow-up 1,8 ± 0,8 (p <0,001)
RAAS-inhibitors								
Choi	2016	Prospective, unrandomised	1213 days	1048	58,9 ± 10,4	59,3 ± 11,1	57,3	Survival (total death): RAAS-i group 0% vs. control group 1,3%. HR 0.015 (95%-CI: 0,001; 47,31) (p = 0,307)

Russell	2007	Prospective, randomised (cross-over)	8 weeks	24	53,5 ± 9,3	N/A	11	Angina pectoris frequency (diary per day): irbesartan period Δ -2,42* vs. placebo period Δ -1,04* (p = ns) Exercise capacity (s): irbesartan Δ -4,83* vs. placebo period Δ 28,6* (p <0,001 in favour of placebo) ** Values is change between baseline and follow-up.*
Calcium channel blockers								
Higuma	2010	Prospective, randomised	12 weeks	37	65,6 ± 9,6	60,9 ± 11,9	54,1	*Nifedipine vs. Diltiazem* – Angina pectoris frequency (diary per week): only available in Figure, no values. Signi-ficant improvement in both groups vs. baseline (p <0,05).
Ogawa	2009	Prospective, unrandomised	1796 days	80	59 ± 9,3	N/A	85,6	Survival (deaths): 0/80 patients (no comparison)
Fukumoto	2008	Prospective, unrandomised	5,2 years	572	62 [31 – 92]	N/A	59,1	Survival (all-cause death): 20/572 patients (no comparison)
Suzuki	2007	Prospective, unrandomised	6 months	15	64,8	N/A	46,7	Angina pectoris frequency (diary per month): only available in Figure, no values. Significant improvement after 6 months (p <0,05)
Chahine	1993	Prospective, randomised	4 weeks	52	55,3 [41 – 71]	55,4 [35 – 67]	58,3	Angina pectoris frequency (diary per day): amlodipine Δ -0,5* vs. placebo Δ -0,12* (p = 0,009) *(no SD reported)* ** Values is change between baseline and follow-up.*
Scholl	1988	Prospective, unrandomised	47 months	48	49 ± 8	N/A	77,1	Survival (all-cause death): 1/48 patients (no comparison)
Egashira	1987	Prospective, unrandomised	4 years	90	58 [38 – 70]	N/A	84,4	Survival (all-cause death): 2/90 patients (no comparison)
Feldman	1982	Prospective, randomised (cross-over)	4 weeks	12	53,3 ± 5,7	N/A	83,3	Angina pectoris frequency (diary per day): baseline 0,35 vs follow-up 0,16 (p = 0,05) *(no SD reported)*

ω-3 fatty acids								
Bozcali	2013	Prospective, randomised	4 months	18	48,8 ± 8,4	47,3 ± 8,5	22,2	Angina pectoris frequency (SAQ): ω-3 fatty acids 81,9 ± 9 vs. placebo 73 ± 15 (p = ns).
Coronary Sinus Reducer								
Tryon	2024	Prospective, unrandomised	120 days	33	54,9 ± 11,0	N/A	33,3	CFR (CFT): baseline 2,1 [[1,95 – 2,30] vs. follow-up 2,7 [2,45 – 2,95] (p = 0,0019)<br>CCS class: baseline 4,0 [3,25 – 4,0] vs. follow-up 2,0 [2,0 – 3,0] (p = <0,001)<br>Quality of life (SAQ): baseline 21,7 ± 15,9 vs. follow-up 54,7 ± 28,4 (p <0,001)
Konigstein	2024	Prospective, unrandomised	4 months	23	65,3 ± 10	N/A	39	CFR (CFT): baseline 1,7 ± 0,5 vs. follow-up 2,9 ± 1 (p = 0,001)<br>Exercise capacity (6-MWT): baseline 303m vs. follow-up 345m (p = 0,04)
Gnan	2023	Prospective, unrandomised	647 days	8	67,8 ± 9,9	N/A	25	CCS class: baseline 2,9 ± 0,6 vs. follow-up 1,5 ± 0,8 (no p value reported)
Aminophylline								
Elliott	1997	Prospective, randomised (cross-over)	3 weeks	10	54 ± 6	N/A	20	Angina pectoris frequency (diary per 3 weeks): aminophylline 11 ± 8 vs. placebo 18 ± 16 (p = ns)<br>Exercise capacity (s): aminophylline 798 ± 149 vs. placebo 765 ± 188 (p = 0,18)
Ranolazine								
Rayner-Hartley	2020	Prospective, unrandomised	6 weeks	31	57 [50 – 67]	N/A	12,9	Angina pectoris frequency (SAQ): baseline 30 [20 – 50], change at follow-up Δ 20 [0 – 50] (p = 0,28)<br>Quality of life (SAQ): baseline 33,3 [25 – 41,7], change at follow-up Δ 33,3 [16,7 – 50] (p < 0,01)

Koh	2020	Prospective, randomised	12 weeks	22	54,6 ± 12,3	51,9 ± 13,9	27,3	Angina pectoris frequency (SAQ): ranolazine Δ 0,70 ± 1,26 vs. placebo Δ 0,27 ± 0,62 (p = 0,53)<br>Exercise capacity (peak work load (W)): ranolazine Δ 0,03 ± 0,27 vs. placebo Δ 0,08 ± 0,12 (p = 0,08)<br>Quality of life (SAQ): ranolazine Δ 0,89 ± 1,49 vs. placebo Δ 1,00 ± 0,99 (p = 0,15)<br>CFR (CAG – Doppler LAD): ranolazine Δ 0,38 ± 0,85 vs. placebo Δ 0,09 ± 0,30 (p = 0,84)
Saha	2017	Prospective, unrandomised	6 weeks	65	49	49,8	41,5	Angina pectoris frequency (SAQ): ranolazine 85,51 vs. conventional 76,03 (p < 0,05)<br>Exercise capacity (METS): ranolazine 6,47 vs. conventional 6,38 (p = 0,397)<br>Quality of life (SAQ): ranolazine 75,7 vs. conventional 70,8 (p < 0,05)
Mehta	2011	Prospective, randomised (cross-over)	10 weeks	10	57 ± 11	N/A	0	Angina pectoris frequency (SAQ): ranolazine 80 [50 – 100] vs. placebo 75 [25 – 75] (p = 0,197)<br>Exercise capacity (DASI): ranolazine 8,6 [3,7 – 11,5] vs. placebo 8,9 [5,4 – 12,1] (p = 0,47)<br>Quality of life (SAQ): ranolazine 75 [60,4 – 83,3] vs. placebo 66,7 [58,3 – 75,0] (p = 0,021)<br>CFR (CMR – MPRI): ranolazine 2,1 [2,2 – 2,4] vs. placebo 1,9 [1,7 – 2,5] (p = 0,66)
Hormone therapy								
Roqué	1998	Prospective, unrandomised	24 hours	15	63 ± 4	N/A	0	CFR (CAG – Doppler LAD): increase in coronary blood flow of 50 ± 30% compared to baseline (p = 0,04)

Allopurinol								
Lim	2018	Prospective, randomised (cross-over)	14 weeks	19	59 ± 10	N/A	42,1	Exercise capacity (min): allopurinol 8,21 ± 1,91 vs. placebo 8,50 ± 2,37 (p = ns)<br>CFR (CAG – Doppler LAD): allopurinol 3,66 ± 0,82 vs. placebo 3,62 ± 1,03 (p = ns)
Phosphodiesterase type 3 inhibitors								
Yoo	2013	Prospective, unrandomised	2 weeks	21	57 ± 8,7	N/A	61,9	Angina pectoris frequency (diary per week): Baseline 3,8 ± 3,1 vs. follow-up 0,5 ± 0,8 (p < 0,001)
Watanabe	2003	Prospective, randomised	6 months	30	58,3 ± 2,9	59,1 ± 3,3	53,3	CFR (CAG – Doppler LAD): Cilostazol 2,85 ± 0,49 vs. placebo 2,37 ± 0,46 (p < 0,01)
Shin	2014	Prospective, randomised	4 weeks	49	51,9 ± 9,4	56,1 ± 8,0	67,3	Angina pectoris frequency (diary per week): cilostazol Δ -3,7 ± 0,5 vs. placebo Δ -1,9 ± 0,6
Nicorandil								
Chen	1997	Prospective, randomised (cross-over)	4 weeks	13	57 ± 6	N/A	76,9	Angina pectoris frequency (diary per 2 weeks): nicorandil 1,08 ± 1,19 vs. placebo 3,23 ± 1,69 (p < 0,001)<br>Exercise capacity (s): nicorandil 443 ± 78 vs. placebo 405 ± 64 (p = 0.036)
Glucose lowering agents								
Suhrs	2019	Prospective, unrandomised	12 weeks	29	67 [62 – 72]	N/A	0	Angina pectoris frequency (SAQ): liraglutide Δ 8,48 vs. placebo Δ -6,10 (p = 0,19)<br>Quality of life (SAQ): liraglitude Δ 1,82 vs. placebo Δ 8,11 (p = 0,11)<br>CFR (TTE – Doppler LAD): liraglitude Δ 0,07 vs. placebo Δ 0,11 (p = 0,58)
Jadhav	2006	Prospective, randomised	8 weeks	33	55,8 ± 8,8	58,1 ± 8,4	0	Angina pectoris frequency (diary per day): metformin 0,16 vs. placebo 0,27 (p = 0,056)
Murakami	1999	Prospective, unrandomised	4 months	10	Not reported	N/A	Not repor.	Angina pectoris frequency (diary per month): baseline 15 ± 17 vs. follow-up 3 ± 3 (p = 0,03)

Renal sympathectic denervation								
Feyz	2020	Prospective, unrandomised	6 months	10	57 ± 11	N/A	90	Angina pectoris frequency (SAQ): baseline 43,3 ± 30,8 vs. follow-up 73,8 ± 32,5 (p = 0,046)<br>Quality of life (SAQ): baseline 32,4 ± 13,5 vs. follow-up 63,1 ± 19,2 (p = 0,023)<br>CCS class: baseline 3,00 ± 0,47 vs. follow-up 1,80 ± 0,92 (p = 0,005)
Vitamin D								
Andishmand	2015	Prospective, unrandomised	2 months	19	51,4 ± 6,5	N/A	15,8	Angina pectoris frequency (diary per day): baseline 0,85 ± 0,29 vs. follow-up 0,61 ± 0,36 (p = 0,003)<br>Exercise capacity (min): baseline 6,9 ± 2,1 vs. follow-up 9 ± 1,9 (p < 0,001)
Transmyocardial laser revascularisation								
Mirhoseini	2008	Prospective, unrandomised	1 year	5	49,7	N/A	Not repor.	CCS class: baseline 3,7 ± 0,4 vs. follow-up 1,5 ± 1,1 (p = not reported)
Endothelin receptor antagonist								
Feenstra	2023	Prospective, randomised (cross-over)	70 days	28	55,3 ± 7,6	N/A	21,4	Angina pectoris frequency (SAQ): macitentan Δ 0,36 ± 12,21 vs. placebo Δ 8,21 ± 21,61 (p = 0,112)<br>Quality of life (SAQ): macitentan Δ 4,61 ± 18,40 vs. placebo Δ 5,21 ± 17,07 (p = 0,906)
Phosphodiesterase type 5 inhibitors								
Denardo	2011	Prospective, unrandomised	45 minutes	23	54 ± 11	N/A	0	CFR (CFT): baseline 2,6 ± 0,5 vs. follow-up 2,8 ± 0,6 (p = 0,06)
Long-acting nitrates								
Kang	2024	Prospective, randomised	4 weeks	20	57 [49 – 59,5]	N/A	75	Angina pectoris frequency (diary per week): baseline 3,0 [1,5 – 4,0] vs. follow-up 0 [0 – 2,0] (no p value reported)

Endothelin A antagonist								
Morrow	2024	Prospective, randomised (cross-over)	12 weeks	118	63,5 ± 9,2	N/A	39,8	Exercise capacity (s): Δ -4,26 at follow-up compared to baseline (p = 0,5871).<br>Quality of life (EQW-5D): Δ -0,007 at follow-up compared to baseline (p = 0,5925)
Statins								
Lee	2017	Prospective, unrandomised	8,1 years	1779	55,4 ± 9,1	55,1 ± 9,9	Not repor.	Survival (all-cause death – % patient-years): statins 0,67% vs. no statins 1,27% (adjusted HR 0,51 [95%-CI; 0,35 – 0,74], p < 0,001)
Kabaklić	2017	Prospective, randomised	6 months	58	63,7 ± 9,6	59,7 ± 9,3	31	Angina pectoris frequency (SAQ): atorvastatin 86,46 ± 12,24 vs. placebo 87,10 ± 14,57 (p = ns)<br>Quality of life (SAQ): atorvastatin 47,50 ± 17,51 vs. placebo 46,45 ± 15,39 (p = ns)
Trimetazidine								
Kim	2018	Prospective, unrandomised	1825 days	882	56,8 ± 11,3	57,2 ± 11,5	59,3	Survival (total death): trimetazidine 1/441 patients vs. no trimetazidine 4/411 patients (HR 0,697 [95%-CI 0,086 – 5,663], p = 0,735)
