# Supplementary material for: Treatment Modalities for Angina with Non-Obstructive Coronary Arteries (ANOCA): A Systematic Review and Meta-Analysis
Source: J Clin Med. 2025 Jun 9;14(12):4069. doi: 10.3390/jcm14124069 (PMC12194334; doi:10.3390/jcm14124069)
Supplement: Supplementary file 1 [file jcm-14-04069-s001.zip › File S3.pdf]

File S3

Secondary outcome – CCS class

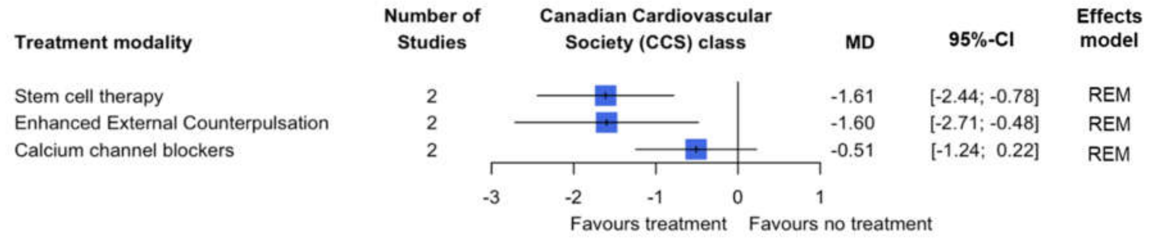

Pooled estimate of the treatment effect on CCS class per treatment modality

Secondary outcome – Changes in coronary blood flow

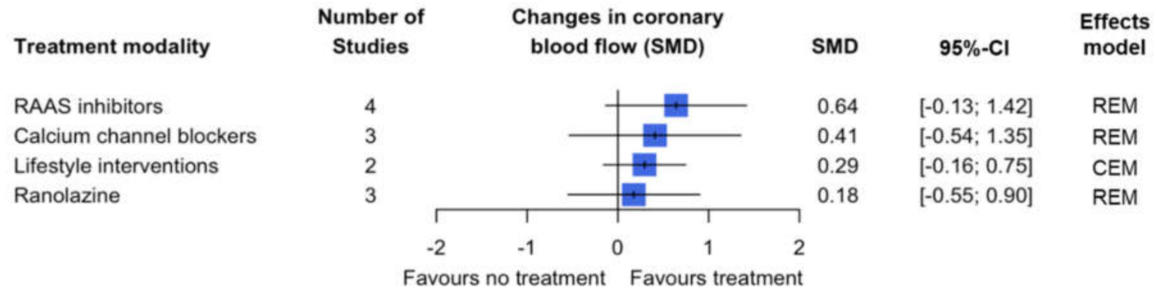

Pooled estimate of the treatment effect on changes in coronary blood flow per treatment modality

Survival

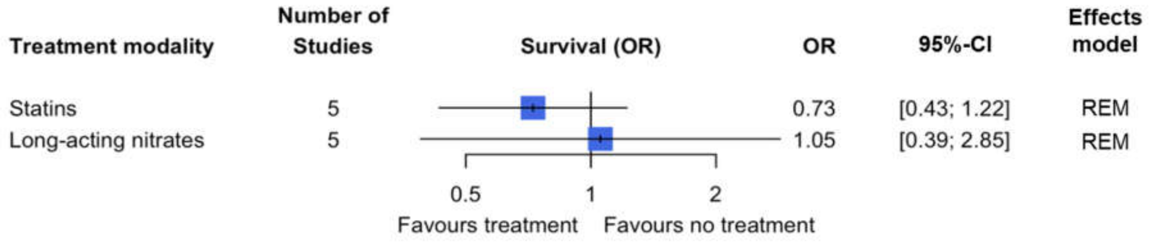

Pooled estimate of the treatment effect on survival per treatment modality
