# Supplementary material for: Treatment Modalities for Angina with Non-Obstructive Coronary Arteries (ANOCA): A Systematic Review and Meta-Analysis
Source: J Clin Med. 2025 Jun 9;14(12):4069. doi: 10.3390/jcm14124069 (PMC12194334; doi:10.3390/jcm14124069)

File S4 - Publication bias assessment

Primary endpoint: Angina pectoris frequency. Egger's test p = 0.0230. Funnel plot:

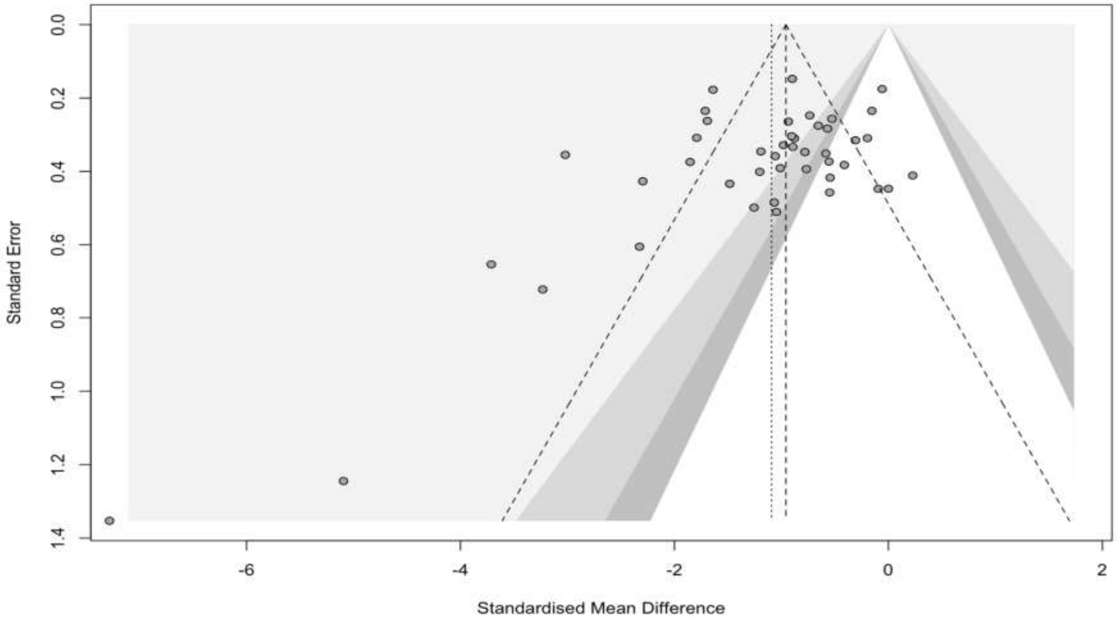

Secondary endpoint: Exercise capacity. Egger's test p = 0.1127. Funnel plot:

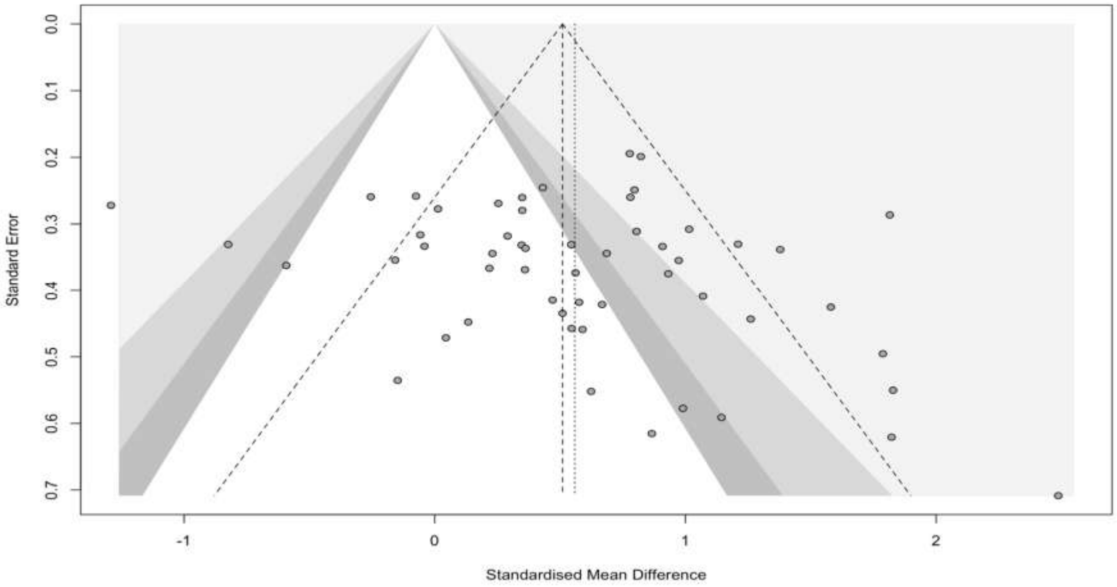

Secondary endpoint: Quality of life. Egger's test p < 0.0001. Funnel plot:

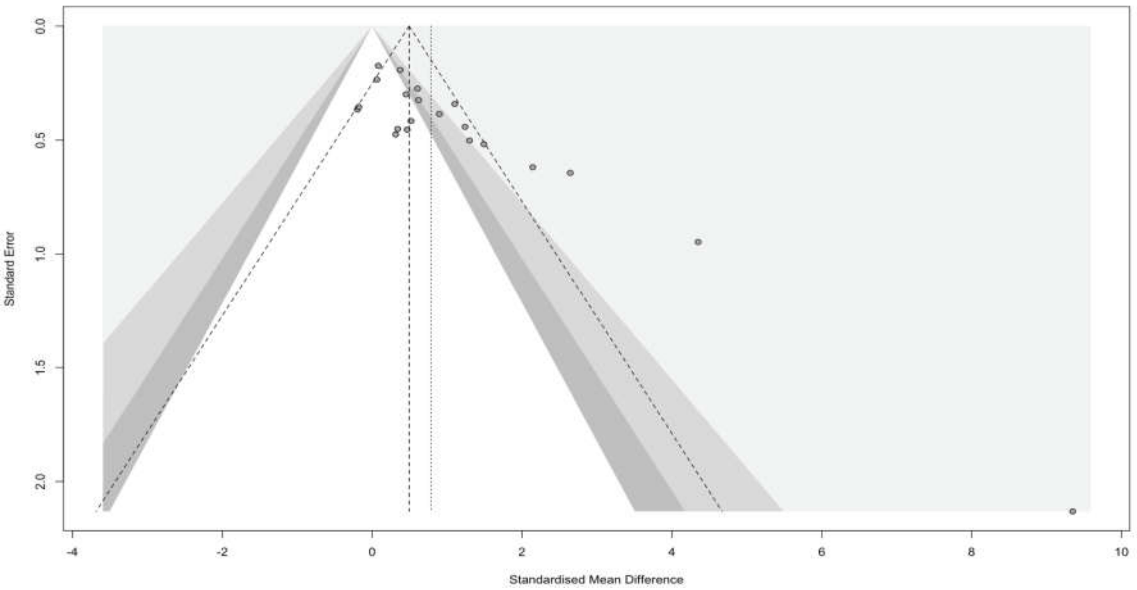

Secondary endpoint: CCS class. Egger's test not possible, too few studies. Funnel plot:

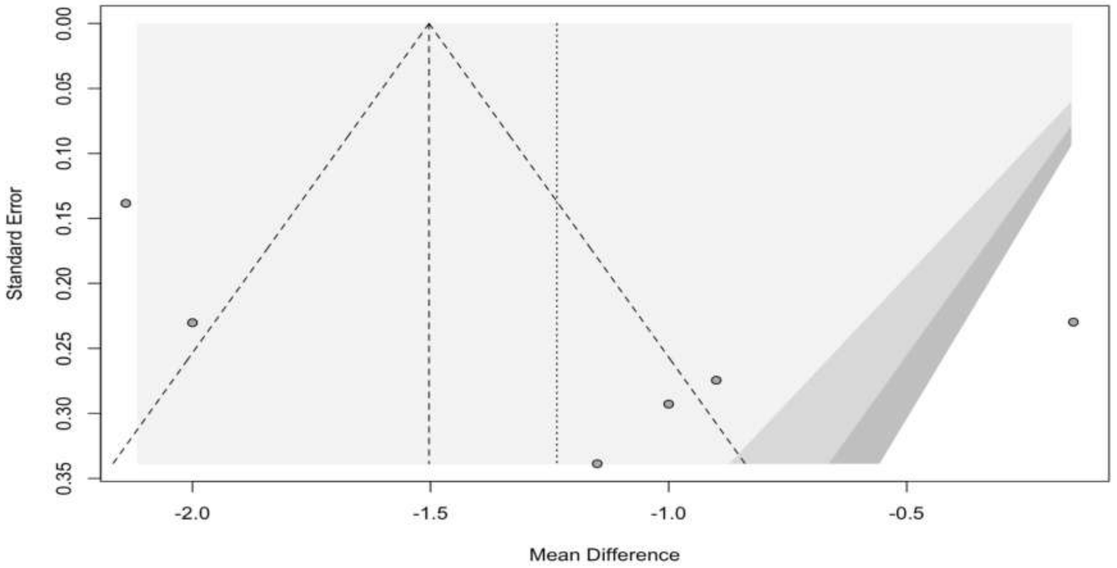

Secondary endpoint: CFR. Egger's test p = 0.0167

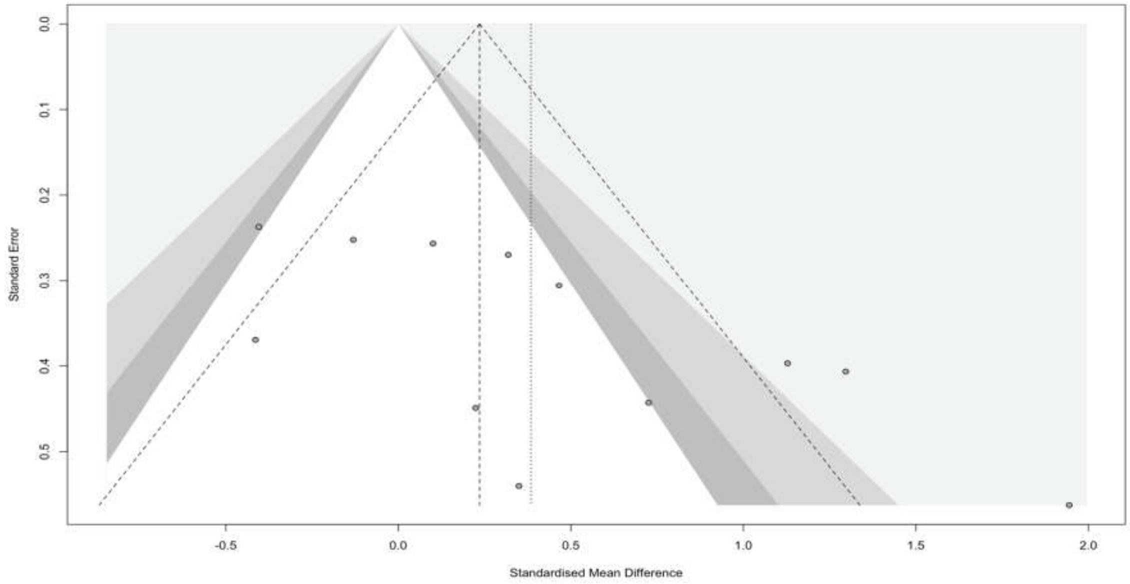

Secondary endpoint: Survival. Egger's test not possible, too few studies. Funnel plot:

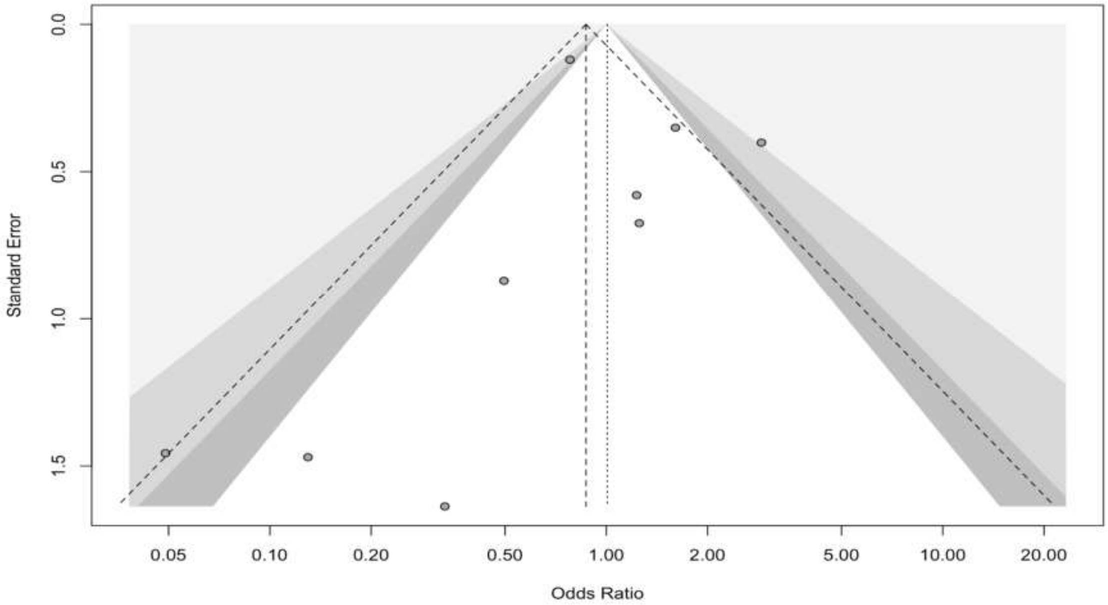

Supplement: Supplementary file 1 [file jcm-14-04069-s001.zip › File S4.pdf]
