# Supplementary material for: Treatment Modalities for Angina with Non-Obstructive Coronary Arteries (ANOCA): A Systematic Review and Meta-Analysis
Source: J Clin Med. 2025 Jun 9;14(12):4069. doi: 10.3390/jcm14124069 (PMC12194334; doi:10.3390/jcm14124069)

File S6 – Subgroup analysis follow-up duration

Primary endpoint: angina frequency – Calcium channel blockers (CEM). Between group difference p = 0.0637

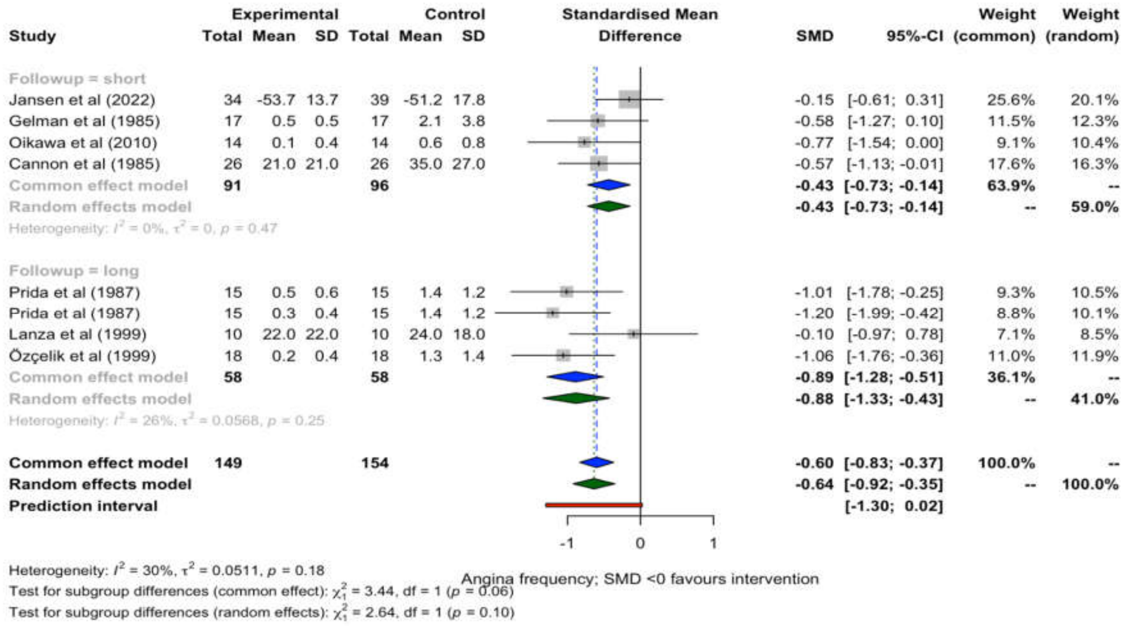

Primary endpoint: angina frequency – Beta-blockers (CEM). Between group difference p = 0.8996

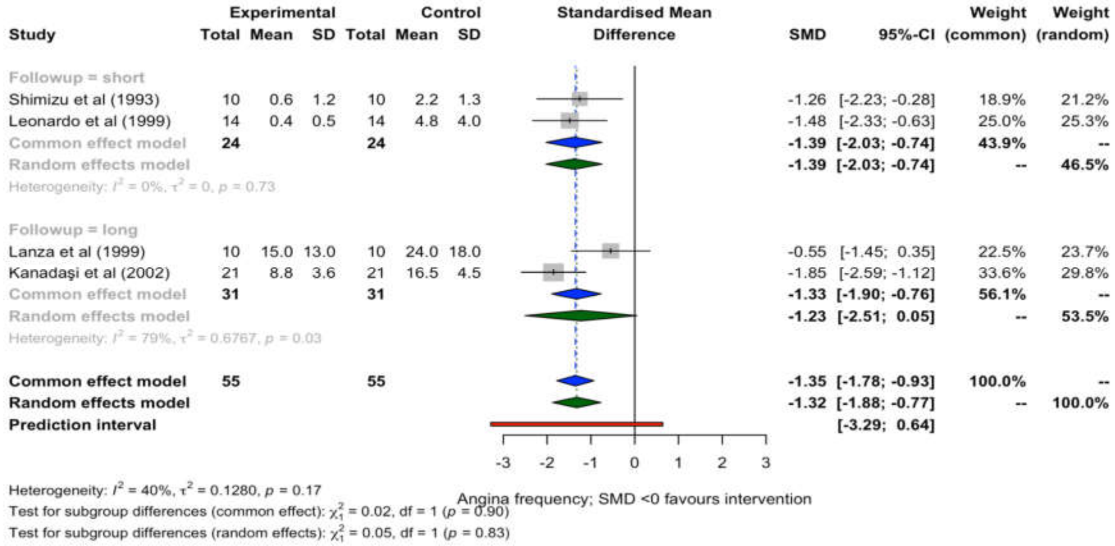

Primary endpoint: angina frequency – Neuromodulation (REM). Between group difference p = 0.0169

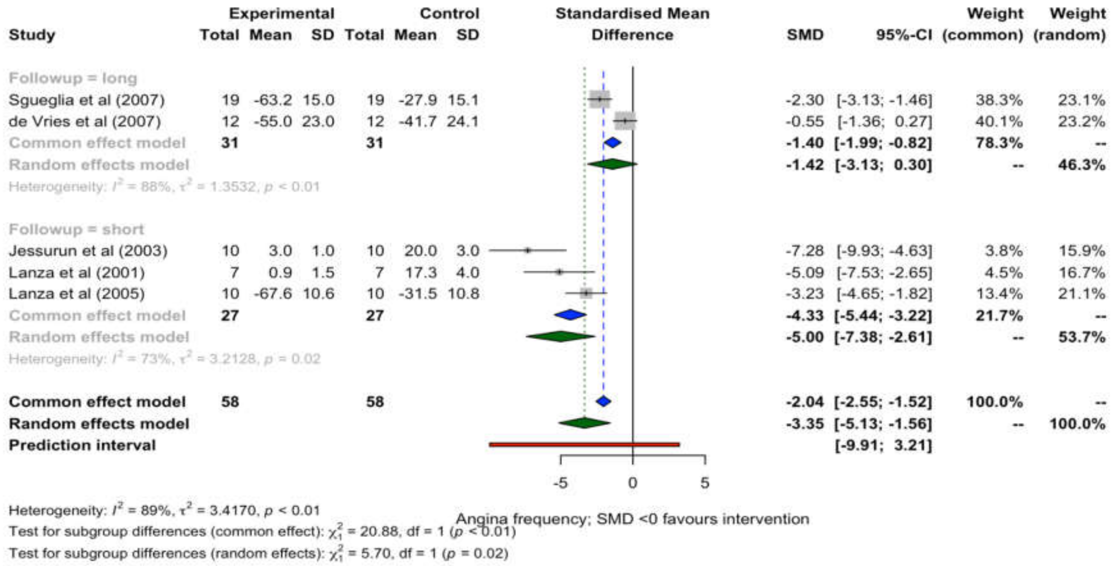

Primary endpoint: angina frequency – Trimetazidine (REM). Between group difference p = 0.9872

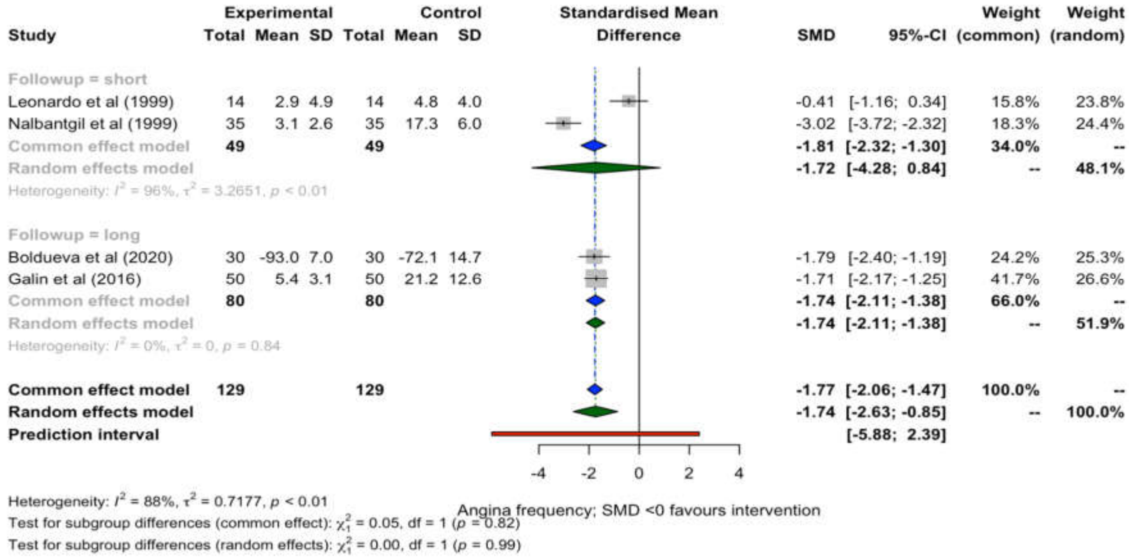

Primary endpoint: angina frequency – Traditional Chinese medicine (REM). Between group difference p = 0.0903

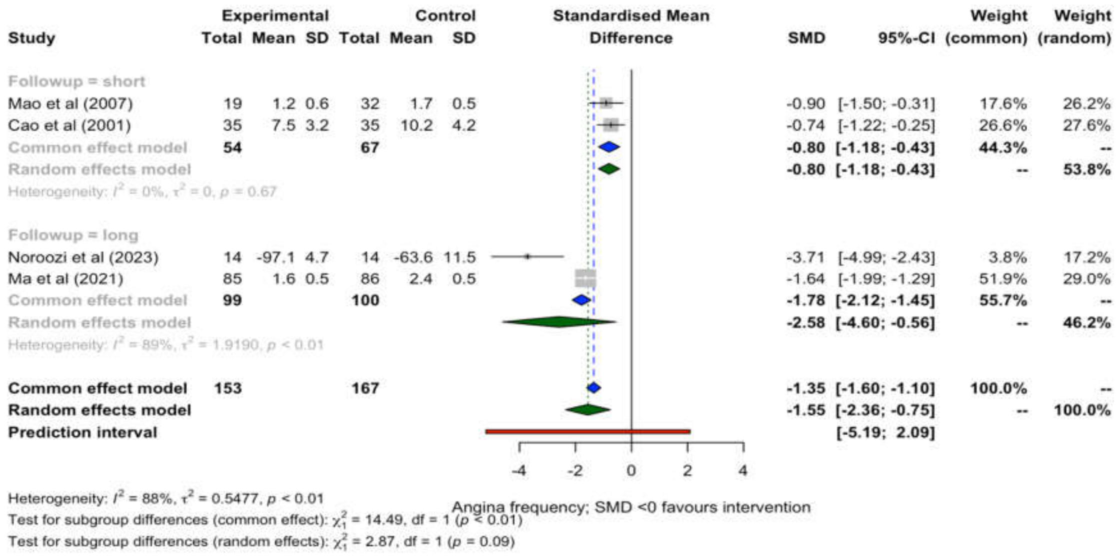

Supplement: Supplementary file 1 [file jcm-14-04069-s001.zip › File S6.pdf]
