# Supplementary material for: Treatment Modalities for Angina with Non-Obstructive Coronary Arteries (ANOCA): A Systematic Review and Meta-Analysis
Source: J Clin Med. 2025 Jun 9;14(12):4069. doi: 10.3390/jcm14124069 (PMC12194334; doi:10.3390/jcm14124069)

File S7 – Subgroup analysis type of study

Primary endpoint: angina frequency – Traditional Chinese medicine (REM). Between group difference p = 0.4782

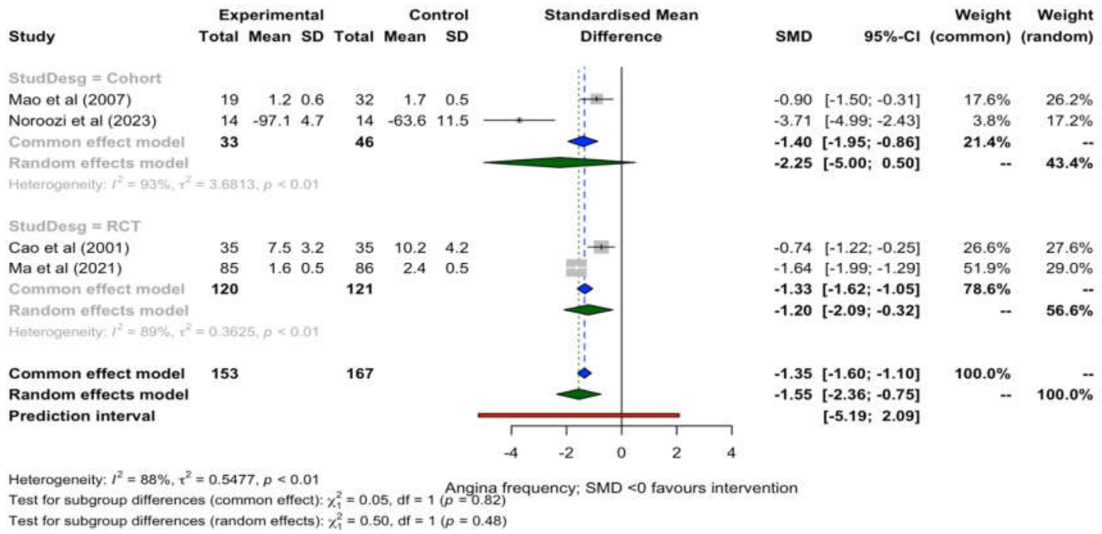

Supplement: Supplementary file 1 [file jcm-14-04069-s001.zip › File S7.pdf]
